# Intralesional flickering in circumscribed choroidal hemangioma: prevalence and association with tumor size

**DOI:** 10.1186/s40942-026-00915-1

**Published:** 2026-08-04

**Authors:** Miri Meira Fogel Levin, Abed Elmajed Haj Yahya, Avner Hostovsky, Iris Moroz, Yael Birger, Ido Didi Fabian, Vicktoria Vishnevskia Dai

**Affiliations:** 1 Goldschleger Eye Institute, Sheba Medical Center, Tel-Hashomer, Ramat Gan, Israel; 2 Gray Faculty of Medical and Health Sciences, Tel Aviv University, Tel Aviv, Israel

**Keywords:** Circumscribed choroidal hemangioma, B-scan ultrasonography, Dynamic ultrasonography, Flickering, Choroidal tumor

## Abstract

**Background:**

Circumscribed choroidal hemangioma (CCH) is a benign vascular choroidal tumor traditionally characterized on ultrasonography by dome-shaped morphology, acoustic solidity, and high internal reflectivity. Dynamic features on real-time B-scan ultrasonography have not been systematically evaluated. This study aimed to characterize the prevalence of intralesional flickering on real-time dynamic B-scan ultrasonography in treatment-naive CCH and to evaluate its relationship to tumor dimensions and exudative activity.

**Methods:**

This retrospective observational study included 29 treatment-naive eyes of 29 patients diagnosed with CCH at a tertiary ocular oncology service between 2015 and 2025. Standardized B-scan ultrasonography at 10 MHz, with real-time documentation of intralesional flickering, was performed per departmental protocol. Tumor largest basal diameter (LBD) and thickness were measured. Spectral-domain optical coherence tomography was reviewed for subretinal and intraretinal fluid. Associations between flickering status and tumor dimensions, exudative fluid, and tumor location were assessed.

**Results:**

Mean age was 55.1 years, 75.9% of patients were male, and 65.5% of tumors were macular. Flickering was identified in 17 of 29 eyes (58.6%). Tumors with flickering had a significantly greater largest basal diameter than tumors without flickering (9.27 versus 6.34 mm; *p* < 0.001) and a trend toward greater thickness (3.00 versus 2.21 mm; *p* = 0.073). Fluid on optical coherence tomography was present in 24 eyes (82.8%) and was associated with greater LBD (*p* = 0.022) and greater tumor thickness (*p* = 0.027). Fluid was more prevalent in eyes with flickering than in those without flickering (94.1% versus 66.7%; *p* = 0.130). Tumor location was not significantly associated with flickering status or fluid presence.

**Conclusions:**

Intralesional flickering on real-time dynamic B-scan ultrasonography is detectable in more than half of treatment-naive CCH and correlates with tumor size, warranting further evaluation as a potential dynamic imaging feature of the lesion.

**Trial registration:**

Not applicable.

**Supplementary Information:**

The online version contains supplementary material available at https://doi.org/10.1186/s40942-026-00915-1.

## Background

Circumscribed choroidal hemangioma (CCH) is a rare, benign vascular hamartoma of the choroid, with a reported male predominance of 62%, most commonly diagnosed between the fourth and sixth decades of life [1, 2]. It typically presents as a solitary orange-red dome-shaped mass located posterior to the equator, with a predilection for macular and juxtapapillary locations [1, 3]. Exudative complications, including subretinal fluid (SRF) and intraretinal fluid (IRF), are reported in up to 84% of symptomatic cases and represent the principal cause of visual impairment and the primary indication for treatment [2, 3].

CCH is a well-recognized diagnostic challenge, accounting for 5% of all posterior uveal pseudomelanomas [4]. In the landmark series of 200 consecutive CCH cases, Shields et al. reported that only 29% had been correctly diagnosed at referral, with choroidal melanoma (29%) and metastasis (9%) being the most common misdiagnoses [3]. Accurate diagnosis relies on multimodal imaging, including ultrasonography (USG), optical coherence tomography (OCT) and indocyanine green angiography [5–10]. On B-scan USG, CCH appears as a dome-shaped, acoustically solid choroidal mass with high internal reflectivity on A-scan [3, 5].

The ultrasonographic phenotype of CCH has historically been characterized in static terms, emphasizing internal reflectivity, acoustic solidity, and lesion morphology [5, 6, 11]. Hemodynamic features, in contrast, have received less systematic attention. Early kinetic ultrasound studies reported no detectable internal vascularization in CCH [12], yet color Doppler imaging has subsequently demonstrated that internal blood flow is present within the lesion [13, 14]. More recently, real-time dynamic B-scan video review has expanded the ultrasonographic characterization of other choroidal lesions, documenting intrinsic vascularity in choroidal nevi and pulsatile flow in vortex vein varices [15, 16]. These observations suggest that real-time dynamic B-scan imaging may capture hemodynamic information within choroidal lesions that is not apparent on conventional static ultrasonography. Whether intralesional flickering occurs in CCH and how it relates to tumor dimensions and exudative activity has not been systematically evaluated.

The purpose of the present study was to characterize the prevalence of intralesional flickering in treatment-naive CCH using real-time dynamic B-scan USG, and to evaluate its relationship to tumor dimensions, exudative fluid, and tumor location.

## Methods

This was a retrospective observational study of consecutive patients diagnosed with CCH and evaluated at the Ocular Oncology Service, Goldschleger Eye Institute, Sheba Medical Center, Israel, between 2015 and 2025. The study adhered to the tenets of the Declaration of Helsinki and was approved by the Institutional Review Board of Sheba Medical Center.

### Study population

Inclusion criteria were: (1) a clinical diagnosis of CCH based on fundoscopic appearance and confirmatory multimodal imaging, established by one of two experienced ocular oncologists (V.V.D. and I.D.F.); (2) B-scan USG performed at our institution prior to any treatment (including photodynamic therapy, cryotherapy, laser photocoagulation, radiotherapy, intravitreal anti-VEGF or corticosteroid injection), with explicit documentation of the presence or absence of intralesional flickering; and (3) spectral-domain optical coherence tomography (SD-OCT) performed at or near the time of USG before treatment. Diffuse choroidal hemangiomas, including those associated with Sturge-Weber syndrome, were excluded.

Of 38 eyes initially identified, nine were excluded: seven had been previously treated at an outside institution, one was excluded because flickering status had not been documented in the USG report, and one lacked pre-treatment OCT data. The final cohort comprised 29 eyes of 29 patients. In every case the diagnosis of CCH rested on its characteristic multimodal imaging phenotype, including high internal reflectivity on standardized A-scan ultrasonography together with the typical indocyanine green and optical coherence tomography features and a benign clinical course over follow-up; tissue biopsy was not performed, in keeping with routine practice for this lesion.

### Clinical examination

Patient demographics were retrieved from the hospital medical records. All patients underwent a comprehensive ophthalmic examination per departmental protocol, including best-corrected visual acuity (BCVA, converted to logMAR for analysis), slit-lamp biomicroscopy, and dilated fundoscopy. Tumor location was classified as macular or extramacular based on fundoscopic documentation and SD-OCT findings. Tumors lying entirely within the temporal vascular arcades were classified as macular. Tumors straddling an arcade were classified as macular if the greater portion of the tumor base lay within the arcades or if the tumor involved or elevated the fovea, and as extramacular otherwise. SD-OCT was performed using the Spectralis HRA + OCT platform (Heidelberg Engineering, Heidelberg, Germany) and reviewed for the presence of SRF and IRF overlying or adjacent to the tumor at presentation. Maximal subretinal fluid height was measured on horizontal and vertical SD-OCT B-scans using the built-in caliper tool as the greatest perpendicular distance between the outer border of the detached neurosensory retina and the apical surface of the retinal pigment epithelium, at the point of maximum elevation whether subfoveal or extrafoveal, and recorded in micrometers.

### Ultrasonographic examination

Standardized ophthalmic USG was performed using the Ellex Eye Cubed system (Ellex Medical, Adelaide, Australia). Per departmental protocol, every ultrasonographic examination of a choroidal lesion followed a standardized sequence regardless of the suspected tumor type. Real-time B-scan imaging was performed first at 10 MHz. Horizontal and vertical sections were obtained through the tumor, and both the basal diameter and thickness were measured in each plane. At least two scans were acquired in each orientation, and the highest value was recorded. Systematic assessment also documented tumor shape, echogenicity, presence or absence of subretinal fluid, and presence or absence of intralesional flickering. A-scan assessment was subsequently performed at 8 MHz to evaluate the internal reflectivity pattern and quantitative reflectivity measurements. All ultrasonographic examinations were performed and interpreted in real time by one of three experienced ophthalmic imaging and retina specialists (I.M., Y.B. and M.F.L.), with findings explicitly recorded in the standardized report.

**Fig. 1:**
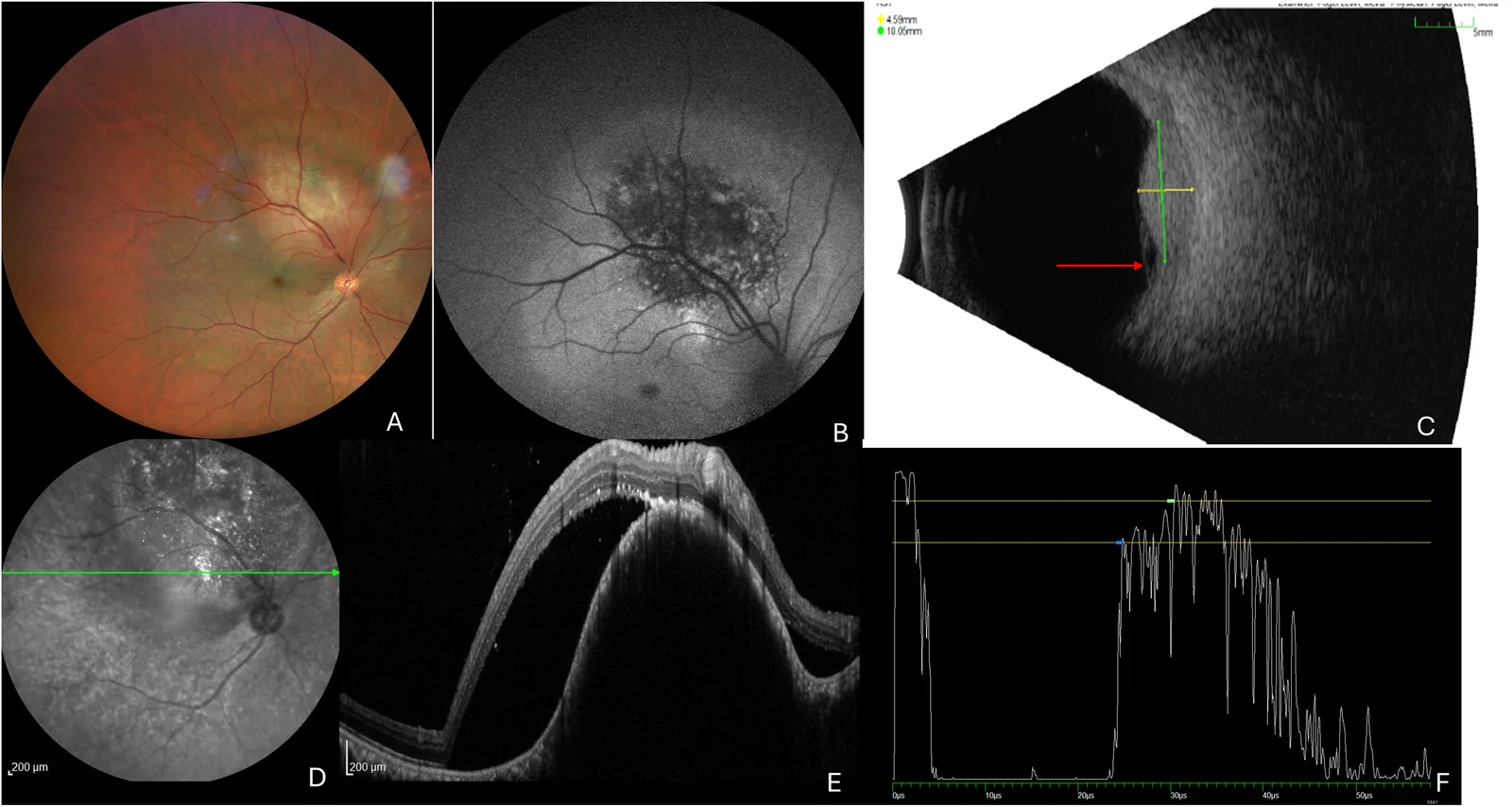
Multimodal imaging of a 39-year-old man with a treatment-naive circumscribed choroidal hemangioma (CCH) of the right eye with intralesional flickering. (**A**) Widefield color fundus photograph demonstrating a hypopigmented, dome-shaped choroidal mass adjacent to the optic disc, extending into the superior macular region. (**B**) Fundus autofluorescence (FAF) imaging revealing a hypoautofluorescent area corresponding to the tumor, surrounded by a ring of hyperautofluorescence representing subretinal fluid. (**C**) B-scan ultrasonography (10 MHz) demonstrating a dome-shaped, acoustically solid choroidal mass with a maximal thickness of 4.59 mm. Subretinal fluid is visible at the margin of the lesion (red arrow). Intralesional flickering was identified during real-time dynamic examination. (**D-E**) Near-infrared reflectance (NIR) image and corresponding spectral-domain optical coherence tomography (SD-OCT) cross-section showing a large dome-shaped choroidal mass with overlying subretinal fluid. (**F**) Standardized A-scan ultrasonography (8 MHz) demonstrating high internal reflectivity, characteristic of choroidal hemangioma

Largest basal diameter (LBD) was defined as the maximal linear dimension of the tumor base in any meridian, and tumor thickness as the maximal apical height perpendicular to the scleral surface. Intralesional flickering was defined as rapid, fluctuating internal echo motion within the tumor substance on real-time dynamic B-scan examination performed with direct scleral contact while the patient fixated a target. The probe was swept back and forth across the lesion to localize the motion and to confirm that it recurred only at the same intralesional point on repeated passes. A finding was recorded as present only when this motion was confined to the tumor substance and absent in the immediately adjacent tissue, reproducible on repeated scanning of the same area, and distinguished from whole-globe cardiac motion. Multimodal imaging of a representative case, including B-scan and A-scan ultrasonography, spectral-domain OCT, fundus autofluorescence, and widefield fundus photography, is presented in Fig. 1.

### Statistical analysis

Continuous variables are presented as mean ± standard deviation, median, and range. Categorical variables are presented as counts and percentages. The Shapiro-Wilk test was used to assess the distribution of continuous variables. Associations between categorical variables (intralesional flickering, tumor location, and presence of fluid) were evaluated using the Fisher exact test. Comparisons of continuous variables between groups defined by flickering status and fluid status were performed using the Mann-Whitney U test. Correlations between continuous variables were assessed using the Spearman rank correlation coefficient. A two-sided p-value of less than 0.05 was considered statistically significant. All statistical analyses were performed using SPSS version 29.0 (IBM Corp., Armonk, NY).

## Results

### Baseline characteristics

Baseline demographic and clinical characteristics are summarized in Table 1. The cohort comprised 29 eyes of 29 patients with a mean age of 55.1 ± 20.3 years (median 60; range 10 to 86). Twenty-two patients (75.9%) were male, and the right eye was affected in 17 cases (58.6%). Systemic comorbidities included hypertension in seven patients (24.1%) and a history of treated malignancy in five (17.2%). Mean BCVA at presentation was 0.54 ± 0.56 logMAR (median 0.35, Snellen equivalent 20/45; range 0.00 to 2.30).

**Table 1:** Baseline demographic and clinical characteristics (*n* = 29)

Characteristic	Value (*n* = 29)
Age, years	
Mean ± SD	55.1 ± 20.3
Median (range)	60 (10–86)
Sex, n (%)	
Male	22 (75.9)
Female	7 (24.1)
Eye, n (%)	
Right	17 (58.6)
Left	12 (41.4)
BCVA at presentation, logMAR	
Mean ± SD	0.54 ± 0.56
Median (range)	0.35 (0.00-2.30)
Systemic comorbidities, n (%)	
Hypertension	7 (24.1)
History of treated malignancy	5 (17.2)
Tumor location, n (%)	
Macular	19 (65.5)
Extramacular	10 (34.5)
LBD, mm	
Mean ± SD	8.07 ± 2.39
Median (range)	8.00 (2.38–14.77)
Tumor thickness, mm	
Mean ± SD	2.67 ± 1.10
Median (range)	2.48 (0.50–5.06)
Intralesional flickering, n (%)	
Present	17 (58.6)
Absent	12 (41.4)
Fluid on OCT, n (%)	
Present	24 (82.8)
Absent	5 (17.2)
Fluid pattern, n	
SRF only	15
IRF only	3
SRF + IRF	6

BCVA = best-corrected visual acuity; LBD = largest basal diameter; SRF = subretinal fluid; IRF = intraretinal fluid; OCT = optical coherence tomography

Nineteen tumors (65.5%) involved the macular region and 10 (34.5%) were extramacular, six of the 19 macular tumors extended to or across a vascular arcade. Mean LBD was 8.07 ± 2.39 mm (median 8.00; range 2.38 to 14.77). Mean tumor thickness was 2.67 ± 1.10 mm (median 2.48; range 0.50 to 5.06). LBD and thickness were strongly correlated (Spearman rho = 0.753, *p* < 0.001). Fluid on OCT was present in 24 of 29 eyes (82.8%); subretinal fluid alone was the predominant pattern (*n* = 15), followed by combined subretinal and intraretinal fluid (*n* = 6) and intraretinal fluid alone (*n* = 3). During follow-up, 20 patients (69.0%) received photodynamic therapy and nine (31.0%) were managed with observation alone.

### Intralesional flickering

Intralesional flickering was identified on real-time dynamic B-scan examination in 17 of 29 eyes (58.6%). Tumors with flickering had significantly larger LBD than those without (9.27 ± 2.06 mm versus 6.34 ± 1.69 mm; Mann-Whitney U, *p* < 0.001) (Table 2). Tumor thickness was also greater in eyes with flickering, though this difference did not reach statistical significance (3.00 ± 1.12 mm versus 2.21 ± 0.93 mm; *p* = 0.073). Representative examples of intralesional flickering on dynamic B-scan USG are shown in Additional file 1.

**Table 2:** Tumor dimensions by flickering status and fluid status

	Present	Absent	*P*
**By flickering status**	**(** ***n*** ** = 17)**	**(** ***n*** ** = 12)**	
LBD, mm	9.27 ± 2.06	6.34 ± 1.69	< 0.001
Thickness, mm	3.00 ± 1.12	2.21 ± 0.93	0.073
**By fluid status**	**(n = 24)**	**(n = 5)**	
LBD, mm	8.55 ± 2.10	5.36 ± 2.35	0.022
Thickness, mm	2.87 ± 1.05	1.72 ± 0.86	0.027

Values are mean ± SD. P values from Mann-Whitney U test. LBD = largest basal diameter; SD = standard deviation

Fluid was present in 16 of 17 eyes (94.1%) with flickering compared with eight of 12 (66.7%) without, though this difference did not reach statistical significance (Fisher exact test, *p* = 0.130). LBD was significantly larger in eyes with fluid compared with those without (8.55 ± 2.10 mm versus 5.36 ± 2.35 mm; *p* = 0.022), and tumor thickness was also significantly greater (2.87 ± 1.05 mm versus 1.72 ± 0.86 mm; *p* = 0.027). Maximal SRF height was measured in the 21 eyes with subretinal fluid (13 with and 8 without flickering) and did not differ between eyes with and without intralesional flickering (median 320 versus 300 μm; *p* = 0.90) and did not correlate with tumor dimensions (largest basal diameter: Spearman rho = -0.14, *p* = 0.59; thickness: rho = 0.36, *p* = 0.13).

Tumor location was not significantly associated with flickering status (*p* = 1.000) or with the presence of fluid (macular 73.7% versus extramacular 100%; *p* = 0.134). BCVA did not differ significantly between eyes with and without flickering (0.58 ± 0.61 versus 0.50 ± 0.51 logMAR; *p* = 0.725).

## Discussion

The principal finding of this study is that intralesional flickering is detectable on real-time dynamic B-scan USG in the majority of treatment-naive CCH, and that its presence is strongly size-dependent. Flickering was identified in 58.6% of eyes overall, and tumors with flickering were substantially larger than those without (LBD 9.27 versus 6.34 mm; *p* < 0.001), with a concordant trend for thickness (3.00 versus 2.21 mm; *p* = 0.073). This size dependence supports the interpretation of intralesional flickering as an observable dynamic imaging correlate of tumor burden in CCH, complementing established structural features such as LBD, thickness, and exudative activity in the multimodal characterization of the lesion.

The origin of the flickering cannot be established from grayscale B-scan, and the following is offered only as a hypothesis. A vascular explanation is plausible: CCH consists of large, thin-walled, blood-filled channels, and color Doppler has demonstrated internal blood flow within these tumors [13, 14]. Slow or sluggish flow within these channels, whether frank flow or the swirling motion of aggregated erythrocytes, could account for the observed motion, as could movement of the many intervascular septa and tissue interfaces within the lesion. Non-vascular and artifactual contributions, however, cannot be excluded. The association with tumor size could reflect a greater volume of vascular tissue in larger lesions, although easier detection of subtle motion in larger tumors may also contribute. Clarifying the mechanism will require prospective acquisition with Doppler correlation and motion correction.

Tumor size was also significantly associated with the presence of exudative fluid, for both LBD (*p* = 0.022) and thickness (*p* = 0.027), consistent with previous reports [17–19]. Flickering and fluid coexisted frequently: fluid was present in 94.1% of eyes with flickering compared with 66.7% of those without, although this difference did not reach statistical significance (*p* = 0.130). The convergence of larger tumor size, detectable flickering, and exudative activity suggests that these features may share a common determinant. Whether flickering contributes to exudation through a direct hemodynamic mechanism, or whether tumor size is the underlying driver of both, cannot be resolved within the present design.

Notably, fluid was present in all 10 extramacular eyes compared with 73.7% of macular eyes. This likely reflects an ascertainment bias rather than a true difference in exudative propensity: extramacular tumors are typically asymptomatic unless exudative fluid extends to the macula, and are therefore less likely to present clinically in the absence of fluid. In contrast, macular tumors may cause symptoms through direct structural disruption even without exudation, allowing detection of fluid-free lesions. This pattern should be considered when interpreting fluid prevalence across tumor locations in clinical cohorts.

Several limitations should be acknowledged. This was a retrospective, single-center study with a relatively small cohort, limiting statistical power and precluding multivariate modeling. All examinations were performed with a single device (Ellex Eye Cubed, 10 MHz) and generalizability to other instruments and settings is unknown. Flickering assessment was based on real-time examination and contemporaneous report documentation, and was classified dichotomously without quantitative grading of amplitude or frequency. A negative examination does not exclude low-amplitude flickering below the resolution of grayscale imaging, and detection is examiner-dependent. The motion was not confirmed by color or pulsed-wave Doppler, so a vascular origin cannot be established from grayscale B-scan alone. In addition, maximal SRF height was measured on the available spectral-domain OCT sections at the point of greatest subretinal elevation rather than by volumetric segmentation, so the value depends on the cross-sections acquired and may not capture fluid outside the sampled scans; total fluid volume, as well as intraretinal and predominantly marginal fluid, is not reflected in this measure. Finally, because flickering may be more readily appreciated in larger lesions, the observed association with basal diameter may be influenced in part by detectability rather than reflecting tumor biology alone. As a tertiary referral center for ocular oncology, the cohort may be enriched for larger or more symptomatic tumors relative to the general CCH population.

## Conclusions

Intralesional flickering on real-time dynamic B-scan USG is a common, size-dependent feature of treatment-naive CCH, detected in nearly 60% of eyes and strongly correlated with largest basal diameter. Intralesional flickering warrants further evaluation as a potential dynamic imaging feature of the lesion. Future prospective studies should evaluate whether intralesional flickering correlates with the volume or distribution of exudative fluid, whether it changes with tumor regression on serial dynamic B-scan imaging before and after treatment, and whether it predicts response to photodynamic therapy, which would establish its utility beyond its association with tumor size.

## Supplementary Information

Below is the link to the electronic supplementary material.

Supplementary Material 1: Additional file 1. Real-time dynamic B-scan ultrasonographic recording demonstrating intralesional flickering in three representative patients with circumscribed choroidal hemangioma (CCH).

## Data Availability

The datasets used and/or analyzed during the current study are available from the corresponding author on reasonable request.

## Abbreviations

BCVA: Best-corrected visual acuity
CCH: Circumscribed choroidal hemangioma
FAF: Fundus autofluorescence
IRF: Intraretinal fluid
LBD: Largest basal diameter
OCT: Optical coherence tomography
SD-OCT: Spectral-domain optical coherence tomography
SRF: Subretinal fluid
USG: Ultrasonography
